# Protective effect of *Curcuma longa* L. extract on CCl_4_-induced acute hepatic stress

**DOI:** 10.1186/s13104-017-2409-z

**Published:** 2017-02-01

**Authors:** Geum-Hwa Lee, Hwa-Young Lee, Min-Kyung Choi, Han-Wool Chung, Seung-Wook Kim, Han-Jung Chae

**Affiliations:** 1 Department of Pharmacology and New Drug Development Institute, Chonbuk National University Medical School, Jeonju, Chonbuk, 561-180 Republic of Korea; 2 CS1 Center, Ottogi Research Center, Ottogi Corporation, Kyeonggi-do, 14060 Republic of Korea; 3 Chemical Genomics National Research Laboratory, Department of Biotechnology, Translational Research Center for Protein Function Control, College of Life Science and Biotechnology, Yonsei University, Seoul, 120-752 Republic of Korea

**Keywords:** Hepatotoxicity, CLL extract, Lipid peroxidation, GSH, Curcumin

## Abstract

**Background:**

The *Curcuma longa* L. (CLL) rhizome has long been used to treat patients with hepatic dysfunction. CLL is a member of the ginger family of spices that are widely used in China, India, and Japan, and is a common spice, coloring, flavoring, and traditional medicine. This study was performed to evaluate the hepatoprotective activity of CLL extract and its active component curcumin in an acute carbon tetrachloride (CCl_4_)-induced liver stress model.

**Methods:**

Acute hepatic stress was induced by a single intraperitoneal injection of CCl_4_ (0.1 ml/kg body weight) in rats. CLL extract was administered once a day for 3 days at three dose levels (100, 200, and 300 mg/kg/day) and curcumin was administered once a day at the 200 mg/kg/day. We performed alanine transaminase (ALT) and aspartate transaminase (AST). activity analysis and also measured total lipid, triglyceride, and cholesterol levels, and lipid peroxidation.

**Results:**

At 100 g CLL, the curcuminoid components curcumin (901.63 ± 5.37 mg/100 g), bis-demethoxycurcumin (108.28 ± 2.89 mg/100 g), and demethoxycurcumin (234.85 ± 1.85 mg/100 g) were quantified through high liquid chromatography analysis. In CCl_4_-treated rats, serum AST and ALT levels increased 2.1- and 1.2-fold compared with the control. AST but not ALT elevation induced by CCl_4_ was significantly alleviated in CLL- and curcumin-treated rats. Peroxidation of membrane lipids in the liver was significantly prevented by CLL (100, 200, and 300 mg/kg/day) on tissue lipid peroxidation assay and immunostaining with anti-4HNE antibody. We found that CLL extract and curcumin exhibited significant protection against liver injury by improving hepatic superoxide dismutase (p < 0.05) and glutathione peroxidase activity, and glutathione content in the CCl_4_-treated group (p < 0.05), leading to a reduced lipid peroxidase level.

**Conclusion:**

Our data suggested that CLL extract and curcumin protect the liver from acute CCl_4_-induced injury in a rodent model by suppressing hepatic oxidative stress. Therefore, CLL extract and curcumin are potential therapeutic antioxidant agents against acute hepatotoxicity.

## Background

Curcumin, the pure active component of *Curcuma longa* L. (CLL) and the yellow pigment that is a characteristic feature of curry [1], has been studied for its anti-inflammatory [2], immunoregulatory [3], and other beneficial effects in models of hepatic dysfunction. Researchers have performed a variety of studies to develop natural products that improve hepatic function, including studies of CLL extracts. The mechanism of the protective effects of CLL against hepatic dysfunction has been suggested to be the inhibition of tumor necrosis factor (TNF)-induced apoptosis [4, 5]. As a polyphenolic antioxidant, curcumin has been suggested to inhibit the activation of fibrosis in vitro by reducing cell proliferation and inducing apoptosis [5]. The antioxidant effects of CLL extracts and curcumin have also been studied in a rat model of carbon tetrachloride (CCl_4_)-induced liver injury [6, 7]. Similarly, the hepatoprotective activity of *Silybum marianum* [8], *Tridax procumbens* [9], *Andrographis paniculata* [10], and *Eucommia ulmoides* [11] have been studied to develop herbal medicines and functional foods to improve hepatic function. The systems used for developing natural products and medicines are usually based upon severe hepatic dysfunction models such as liver necrosis, necrosis, and cirrhosis [12]. However, people are frequently exposed to subclinical hepatic stress, and, if a hepatotoxin-associated hepatic event exceeds the hepatic capacity for clearing these toxins, hepatic function can be transiently decreased, leading to acute liver failure. Chemicals often cause subclinical injury to the liver that manifests only as abnormal liver enzyme levels, such as AST. Drug-induced liver injury is responsible for 5% of all hospital admissions and 50% of all cases of acute liver failure [13]. Although treatment of acute liver failure is considered clinically important [14, 15], few preventive products or medicines have been established. In this study, we evaluated the hepatoprotective activity of CLL extract and curcumin in a CCl_4_-induced acute liver toxicity rat model. Compared with other studies of CCl_4_-induced liver cirrhosis, fibrosis and other severe hepatic toxicities [16–19], our research design is an acute/transient toxicity study based on “a low dose of chemical toxin and one time exposure without histological abnormalities.” Considering that transient or acute toxicity can be more frequently happening to human, the acute/transient toxicity model might have a more importantly clinical meaning than other chronic/severe toxicity. We also sought to determine whether the antioxidant properties of CLL extracts or curcumin are involved in the protective effects against acute liver toxicity.

## Methods

### Extract preparation

Korean CLL rhizomes were harvested from Jindo, Korea and extracts were manufactured by the Ottogi Corporation (Seoul, Korea). The roots were crushed and loaded into an extractor. The first and second extractions were carried out with 50% ethanol. The second extracts were filtered and gathered with the first extracts and were concentrated under reduced pressure (20 brix). Twenty percent dextrin was added to the final extracts and they were sterilized. The total yield was 18% based upon weight.

### Quantitation of curcumin and curcuminoids using HPLC–DAD

Curcumin and curcuminoids were prepared with 100% methanol and quantitated with an HPLC system (Agilent 1100 series, Germany) equipped with a Zorbax Eclipse C18 column (250 × 4.0 mm^2^). The mobile phase consisted of 1% acetic acid in water (A) and 52% acetonitrile (B); the column was equilibrated for 10 min in the mobile phase and then washed with 100% (B) for 10 min. The column was operated at room temperature with a 1 ml/min flow rate. The injection volume was 5 μl, and curcumin, curcuminoids, bis-demethoxycurcumin (BDMC), and demethoxycurcumin (DMC) were detected at a wavelength of 424 nm.

### Reagents and chemicals

Carbon tetrachloride and curcumin were purchased from Sigma Chemicals Co. (St. Louis, MO, USA). The commercial kits used for assaying liver enzymes and antioxidants are described below.

### Animals

One hundred male Sprague–Dawley (SD) rats (250–280 g) were purchased from Central Lab Animal Inc. (Seoul, Korea) randomly assigned into groups. The experimental animals were given free access to standard diet and water in rooms maintained at 25 °C on 12-h light/dark cycles. Four rats were sacrificed for preliminary CCl_4_ toxicity testing, and 12 of the surviving rats were placed in each of the following groups. Control rats were injected with olive oil (vehicle; 0.1 ml/kg body weight, i.p.) for 72 h. The curcumin group was intragastrically administered with 200 mg/kg curcumin for 72 h. The CLL group was intragastrically administered with 300 mg/kg CLL extract. The CCl_4_ group received CCl_4_ (0.1 ml/kg, i.p.) for 72 h. The CCl_4_-curcumin group received curcumin (200 mg/kg) intragastrically before 3 days of CCl_4_ treatment. The CCl_4_-CLL groups received 100 mg/kg, 200 mg/kg, or 300 mg/kg of CLL extract intragastrically before 3 days of CCl_4_ treatment. All of the experimental protocols conducted on rats were performed in accordance with internationally accepted principles for laboratory animal use and care and approval was obtained from the Care and Use of Laboratory Animals Committee of Chonbuk National University Hospital. All procedures were also approved by the Institutional Animal Care and Use Committee of Chonbuk National University Hospital (IACUC protocol CBU 150608-25).

### Liver immunohistochemistry

Immunohistochemistry was performed using 4-μm-thick deparaffinized liver tissue sections as described earlier [20]. Briefly, deparaffinized liver slices were incubated overnight with antibodies against 4-hydroxynonenal (4-HNE). Antibody detection was performed using the DAKO EnVision + System Peroxidase/DAB kit.

### AST and ALT activity

Serum levels of liver enzymes AST and ALT were estimated using commercially available diagnostic kits (Cat. AM101-K, Asan Pharm, Seoul, Korea) according to the manufacturer’s protocol.

### Lipid profile analysis

Total cholesterol, high-density lipoprotein (HDL) cholesterol, low-density lipoprotein cholesterol (LDL), and triglycerides (TG) were estimated using commercially available diagnostic kits (Cat. AM203-K and AM 202-K, Asan Pharm).

### Antioxidant enzymes

The activity of SOD and glutathione peroxidase (GPx) was analyzed using assay kits from Cayman according to the manufacturer’s instructions (Cat.706002 and 703102, Cayman, Ann Arbor, MI, USA).

### GSH/GSSG analysis

The level of serum reduced and oxidized glutathione (GSH and GSSG) was measured using a kit from BioVision (Cat. K264, BioVision, Inc, CA, USA) according to the manufacturer’s protocol.

### Determination of tissue lipid peroxidation

The level of serum and liver lipid peroxidation was measured using a thiobarbituric acid reactive substances (TBARS) kit from Cayman (Cat. 10009055) according to the manufacturer’s protocol.

### Statistics

Results are presented as the mean ± SEM. MicroCal Origin software (Northampton, MA, USA) was used for all statistical calculations. Differences were tested for significance using one-way analysis of variance (ANOVA) with Duncan’s multiple range test. Statistical significance was set at *p* < 0.05.

## Results

### Curcuminoid components of CLL turmeric

**Fig. 1:**
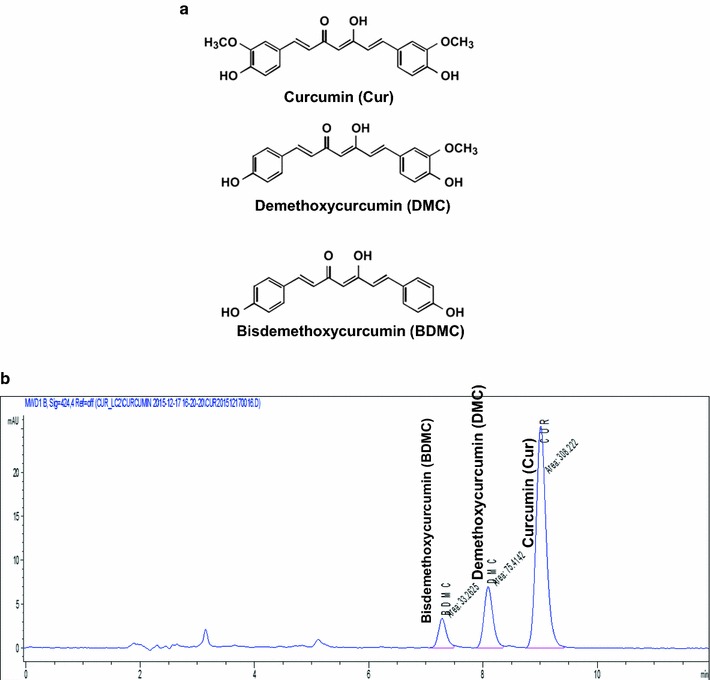
HPLC analysis of CLL extract. **a** Chemical structure of curcumin, demethoxycurcumin (DMC), and bisdemethoxycurcumin (BDMC). **b** HPLC chromatogram analysis of CLL extract

From the CLL extract, major compounds including curcumin, bis-demethoxycurcumin (BDMC), and demethoxycurcumin (DMC) (Fig. 1a) were identified and quantified using HPLC. From 100-g CLL extracts, curcumin (901.63 ± 5.37 mg/100 g), BDMC (108.28 ± 2.89 mg/100 g), DMC (234.85 ± 1.85 mg/100 g) and total curcuminoids (1244.76 ± 3.86 mg/100 g) were quantified (Table 1). The representative HPLC chromatogram is presented in Fig. 1b.

**Table 1:** Analysis of CLL extract with HPLC

Component	mg/100 g
Curcumin	901.63 ± 5.37
Curcuminoids	1244.76 ± 3.86
BDMC	108.28 ± 2.89
DMC	234.85 ± 1.85

The values are shown as mean ± SEM (n = 3)

### CLL turmeric extract and curcumin protect against the CCl_4_-induced toxicity profile

**Fig. 2:**
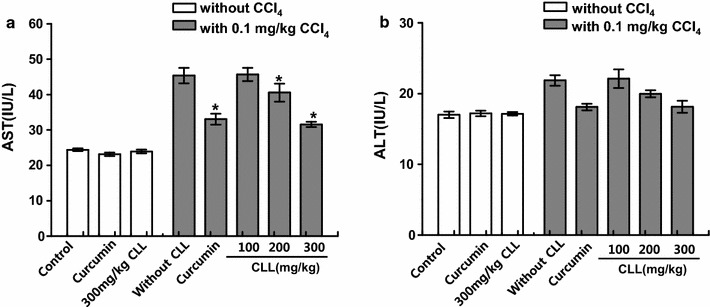
Effects of CLL extract on serum liver biomarkers. Serum AST (**a**) and ALT (**b**) were analyzed in control, 0.1 ml/kg CCl_4_, CCl_4_ with curcumin, CCl_4_ with CLL extract (100, 200, or 300 mg/kg), 200 mg/kg curcumin alone, and 300 mg/kg CLL extract alone groups. *Each bar* represents the mean value of experiments performed in triplicate ± S.E.M. (*n* = 10). *p < 0.05 compared with the CCl_4_ group

To examine the role of CLL turmeric extract and curcumin in hepatic toxicity, the extract and curcumin were applied to a CCl_4_-induced acute toxicity model. The serum activity of AST was increased in the CCl_4_ group compared with the control group (Fig. 2). CCl_4_-increased AST activity was significantly reduced in the presence of CLL extract or curcumin. Although serum ALT level showed a similar pattern to AST level, there were no significance differences between CCl_4_ and the combined CLL extract or curcumin groups. Consistent with these data, AST serum level rather than ALT has been used as a biochemical marker for early acute hepatotoxicity [21, 22].

### CLL turmeric extract does not affect serum lipid profile in a CCl_4_-induced acute toxicity model

Serum cholesterol, triglycerides, and LDL levels did not vary significantly between the CCl_4_ group and the control group. Administration of CLL extract alone resulted in a non-significant change in lipid profiles compared to the control group (Table 2).

**Table 2:** Effect of CLL extract on TG, total-cholesterol, LDL-cholesterol, and HDL-cholesterol at CCl_4_-induced acute stress in rats

Groups	Serum levels (mg/dl)			
	TG	Total-cholesterol	LDL-cholesterol	HDL-cholesterol
Control	55.15 ± 4.98	99.84 ± 2.30	59.50 ± 6.35	48.40 ± 1.92
Curcumin	57.25 ± 4.15	101.2 ± 11.55	63.25 ± 3.43	50.80 ± 1.25
CLL Ex	60.5 ± 1.15	105.37 ± 11.25	67.28 ± 2.08	51.18 ± 4.5
CCl_4_	61.29 ± 0.98	103.29 ± 9.92	63.19 ± 3.75	49.25 ± 3.95
CCl_4_-100 mg/kg CLL	61.2 ± 1.02	100.83 ± 10.29	59.8 ± 6.15	47.28 ± 5.67
CCl_4_-200 mg/kg CLL	58.9 ± 2.13	99.5 ± 11.5	64.55 ± 3.82	45.56 ± 4.95
CCl_4_-300 mg/kg CLL	54.9 ± 4.12	103.38 ± 11.39	56.5 ± 9.55	45.72 ± 5.57
CCl_4_-curcumin	57.65 ± 5.15	105.25 ± 9.22	60.29 ± 4.89	45.68 ± 5.25

### CLL turmeric extract increases antioxidant enzymes in a CCl_4_-induced acute toxicity model

**Fig. 3:**
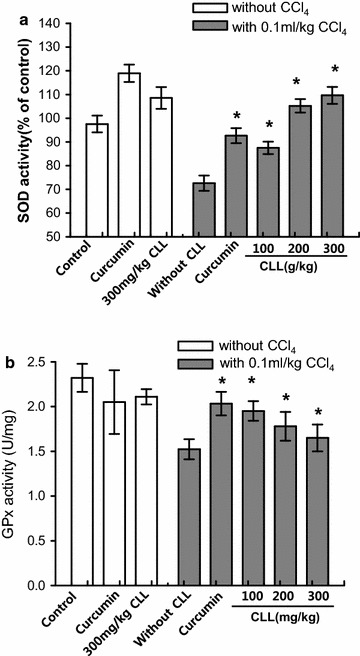
Effects of CLL extract on SOD and GPx activity. **a** Hepatic SOD and **b** GPx activity was analyzed in control, 0.1 ml/kg CCl_4_, CCl_4_ with curcumin, CCl_4_ with CLL extract (100, 200, or 300 mg/kg), 200 mg/kg curcumin alone, and 300 mg/kg CLL extract alone groups. *Each bar* represents the mean value of experiments performed in triplicate ± S.E.M. (*n* = 10). *p < 0.05 compared with the CCl_4_ group

Liver activity of SOD and GPx was decreased in the CCl_4_ group compared with the control group (Fig. 3a, b). The CCl_4_-induced decrease in SOD and GPx activity recovered after treatment with CLL extract or curcumin. In the SOD activity analysis, CLL extract showed a dose-dependent recovery effect; the highest CLL extract dose, 300 mg/kg, showed the greatest protective effect against decreased SOD activity. In the GPx activity analysis, a relatively low dose of 100 mg/kg CLL showed the greatest recovery effect against decreased GPx activity, similar to the recovery effect of curcumin.

### CLL turmeric extract inhibits lipid peroxidation in a CCl_4_-induced acute toxicity model

**Fig. 4:**
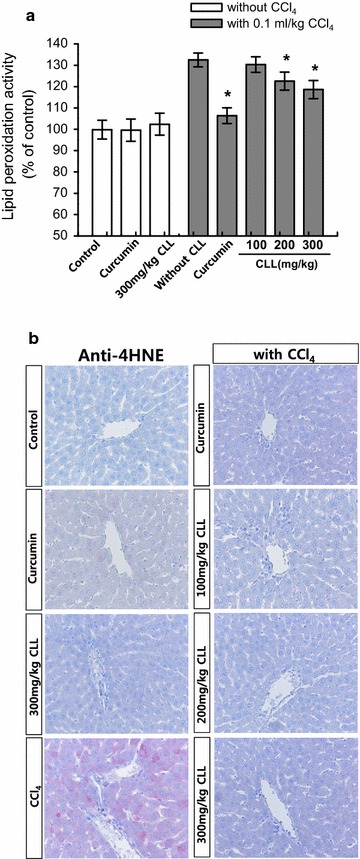
Effects of CLL extract on hepatic lipid peroxidation. The formation of **a** malondialdehyde (MDA) and **b** 4-HNE was analyzed in control, 0.1 ml/kg CCl_4_, CCl_4_ with 200 mg/kg curcumin, CCl_4_ with CLL extract (100, 200, or 300 mg/kg), 200 mg/kg curcumin, and 300 mg/kg CLL extract groups. *Each bar* represents the mean value of experiments performed in triplicate ± S.E.M. (*n* = 10). *p < 0.05 compared with the CCl_4_ group

Increased levels of reactive oxygen species (ROS) induce membrane lipid peroxidation and the production of associated by-products such as malondialdehyde (MDA) and 4HNE [23]. As a product of lipid peroxidation, the MDA level can reflect the liver lipid peroxidation level [24]. In damaged tissues, 4HNE has been found in higher quantities during oxidative stress due to the increase in the lipid peroxidation chain reaction [25]. Figure 4a shows that the liver MDA level increased significantly in CCl_4_-treated mice compared with the control group. Treatment with CLL extract (200 and 300 mg/kg) reduced this CCl_4_-induced increase in MDA. In addition, our results showed that the number of 4-HNE-stained hepatocytes increased in the CCl_4_-treated mice (Fig. 4b). Treatment with CLL extract (100, 200, and 300 mg/kg) reduced the CCl_4_-induced increase in 4-HNE-stained cells. Curcumin treatment consistently showed similar effects to CLL extract. These data collectively suggest that CLL extract protects the liver against CCl_4_-induced oxidative stress.

### CLL turmeric extract and curcumin affect GSH profiles in a CCl_4_-induced acute toxicity model

**Fig. 5:**
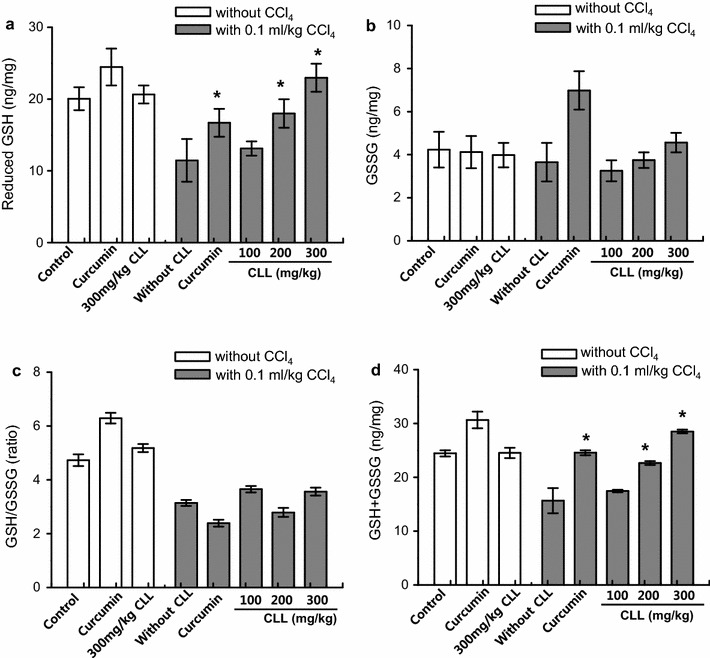
Effects of CLL extract on hepatic redox capacity. **a** Reduced glutathione (GSH), **b** total GSH, **c** GSH/oxidized glutathione (GSSG) ratio and **d** GSH+GSSG content were analyzed in control, 0.1 ml/kg CCl_4_, CCl_4_ with 200 mg/kg curcumin, CCl_4_ with CLL extract (100, 200, or 300 mg/kg), 200 mg/kg curcumin, and 300 mg/kg CLL extract groups. *Each bar* represents the mean value of experiments performed in triplicate ± S.E.M. (*n* = 10). *p < 0.05 compared with the CCl_4_ group

GSH is an important cellular antioxidant that protects cells against ROS-induced liver injury [26]. The efficient transformation of GSH to GSSG has been suggested to be a marker of redox capacity to explain the cellular redox environment [27, 28]. The data shown in Fig. 5a confirm the decrease in GSH level in CCl_4_-treated rats. CLL extract dose-dependently rescued the CCl_4_-induced decrease in liver GSH level. The oxidation product, GSSG, showed a similar pattern (data not shown). Compared to the control group, the level of total glutathione, including GSSG, was significantly reduced by CCl_4_ (Fig. 5b). CLL extract significantly recovered the decreased total GSH level in a dose-dependent manner. The recovery effect of 300 mg/kg CLL was similar to that of curcumin, suggesting that CLL extract and its pure component curcumin protect against oxidative stress by enhancing the intrahepatic redox capacity.

## Discussion

In this report, we showed that the application of CLL extracts markedly inhibited acute hepatic failure in a rat model of acute hepatic injury, which was induced at least in part by free radical formation. Our data suggest that CLL and its active component curcumin are effective against acute liver stress by enhancing redox capacity and antioxidant enzyme activity. In this study, the acute CCl_4_ toxicity model was used to evaluate the efficacy of CLL extract in acute hepatic stress. Acute CCl_4_ administration is a widely used experimental model that mimics the acute liver failure caused by toxic substances [29–31]. Although the liver plays a key role in transforming and clearing chemicals, certain medicinal agents, when taken in excess, may injure the organ. Other chemical agents or industrial agents can also induce hepatotoxicity. These chemicals often cause subclinical injury to the liver, increasing liver enzymes, but not causing pathological abnormalities such as histological and hepatosteatosis status changes. Drug-induced acute liver failure is an important clinical issue [13], and thus therapeutic and preventive strategies against acute liver toxicity need to be developed. In the acute toxicity model, CCl_4_ did not induce hepatic lipid accumulation (Table 2), but did increase activity of the main liver enzyme AST (Fig. 2), a routinely observed acute toxicity-associated clinical symptom. CLL extract inhibited this increase in AST activity. Although AST and ALT aminotransferases are both highly concentrated in the liver, this model showed an increase in AST, but not ALT. A similar pattern was reported recently in cases of acute hepatic and transient stress [22, 32]. AST is localized in the mitochondria, whereas ALT is distributed throughout the cytoplasm. In the case of hepatic stress, mitochondrial damage with ROS accumulation tends to increase the level of AST rather than ALT [33, 34]. The manifestation of acute hepatotoxicity in acute stress is highly variable, ranging from asymptomatic elevation of liver enzymes to fulminant hepatic failure [35]. Because of the considerable disease burden, there is growing interest in understanding acute hepatic failure. One representative system for understanding acute hepatic failure is the CCl_4_ model. Metabolic activation of CCl_4_ by mixed function oxidases is required to induce hepatotoxicity. The initial step in liver injury induction by CCl_4_ is mainly its dehalogenation by cytochrome p-450 2E1 (CYP2E1) to a trichloromethyl free radical (${\text{CCl}}_{3}^{ - }$), which leads to hepatic toxicity [36]. A single injection of CCl_4_ did not induce hepatic lipid accumulation, but simply resulted in increased AST activity and hepatic ROS accumulation. The damage pattern was transient, with recovery to a normal state (data not shown). The main point of this study was transient stress. Hepatic stress that occurs during life is typically transient; at rest, the liver recovers after a short period of time and therefore the stress often goes unnoticed clinically. The initial hypothesis of this study was that foods that prevent routine acute stress might be good for maintaining hepatic health; in this regard, CLL extract was a strong candidate for testing the hypothesis. Curcumin and CLL extracts have been frequently studied with regard to hepatoprotective function. However, the main design of those studies was based on a high level of toxin-induced hepatic dysfunction [2, 37, 38], which varied from the present study’s design; acute or transient toxicity without abnormal liver function except liver enzyme. Throughout this study, CLL extract and its active component curcumin showed antioxidant enzyme activity and a regulatory effect against the accumulation of ROS, including lipid peroxidation, resulting in a protective effect against CCl_4_-induced acute hepatotoxicity. Although the suggested antioxidant mechanism is similar to its application in severe hepatotoxicity, this study strongly suggests the possible application of CLL to transient or acute hepatic stress conditions. Other studies indicated that CLL treatment induces an augmentation of hepatic Nrf-2 protein levels [2] and stimulates antioxidant activity [38–40]. Curcumin, the main component of CLL, significantly reduced the CCl_4_-induced increase in hepatic MDA [7], implying that CLL exerts protective effects against CCl_4_-induced liver damage by preventing lipid peroxide formation and by blocking the oxidative chain reaction [41]. Our results indicated that CCl_4_ administration led to a marked depletion of glutathione (GSH) level in the liver. GSH, a cytosolic tripeptide, is ubiquitously present in all cell types at millimolar concentrations and is the major non-enzymatic regulator of intracellular redox homeostasis [42]. GSH is oxidized to GSSG by the enzymatic reaction catalyzed by glutathione peroxidase (GPx), which is reduced back to GSH by glutathione reductase [43]. Our results showed a drastic reduction in the activity of GPx in the liver caused by CCl_4_, which could compromise the defenses of the liver. Our results suggested that CCl_4_ administration decreased the concentrations of reduced GSH and total GSH in the liver, altering the redox status of the cells, and that treatment with CLL extract and its active component curcumin led to recovery of redox balance. Increases in the levels of reduced GSH and total GSH upon treatment with CLL extracts may be involved in the protective mechanism against CCl_4_-induced liver toxicity.

## Conclusion

In this study, CLL extract and its active component curcumin showed protective activity against CCl_4_-induced hepatotoxicity. In addition to antioxidant activity, CLL extracts and their active components might play a role in restoring the liver redox capacity, as represented by GSH-GSSG cycling capacity. In future studies, derivatives of CLL extracts should be examined in various experimental models of acute toxicity in addition to CCl_4_. Active derivatives may be potential drug candidates for acute liver failure and toxicity.

## Abbreviations

CLL: *Curcuma longa* L. turmeric
CCl_4_: carbon tetrachloride
AST: aspartate transaminase
ALT: alanine transaminase
MDA: malondialdehyde
SOD: superoxide dismutase
GPx: glutathione peroxidase
4-HNE: 4-hydroxynonenal
ROS: reactive oxygen species
CYP2E1: cytochrome p-450 2E1
BDMC: demethoxycurcumin
DMC: demethoxycurcumin
LDL: low-density lipoprotein
HDL: high density lipoprotein
