# Editorial expression of concern: Protective effect of *Curcuma longa* L. extract on CCl4-induced acute hepatic stress

**DOI:** 10.1186/s13104-024-06818-4

**Published:** 2024-06-11

**Authors:** Geum-Hwa Lee, Hwa-Young Lee, Min-Kyung Choi, Han-Wool Chung, Seung-Wook Kim, Han-Jung Chae

**Affiliations:** 1 Department of Pharmacology and New Drug Development Institute, Chonbuk National University Medical School, Jeonju, 561-180 Chonbuk Republic of Korea; 2 CS1 Center, Ottogi Research Center, Ottogi Corporation, Kyeonggi-do, 14060 Republic of Korea; 3 Chemical Genomics National Research Laboratory, Department of Biotechnology, Translational Research Center for Protein Function Control, College of Life Science and Biotechnology, Yonsei University, Seoul, 120-752 Republic of Korea

The Editor is issuing this Editorial Expression of Concern to inform readers that concerns have been raised about some of the data reported in this article [1]. Panels Con Anti-4HNE, 4w CCI4 Anti-4HNE, Curcumin with CII4, and Curcumin Anti-4HNE of Fig. 4b each appear to overlap with panels that were published in an article with common authors [2], which was under review simultaneously to this article. Several of these images were described differently in these two publications. After these concerns were raised, a correction was issued to [2] that removed these duplicating images from that article.

Han-Jung Chae has stated on behalf of all authors that they agree with this Editorial Expression of Concern.
